# Improving the Quality of Lyophilized Animal Vaccines: A Review of Optimization Strategies for Freeze-Drying Processes

**DOI:** 10.3390/vetsci13090972

**Published:** 2026-09-16

**Authors:** Fangjing Huang, Jiajie Jiao, Zexi Wang, Hongying Jie, Jiahao He, Xiaoxin Zuo, Yanhong Zhao, Yu Lu, Jianhong Gu, Fang Lyu

**Affiliations:** 1 College of Veterinary Medicine, Yangzhou University, Yangzhou 225009, China; mx120231052@stu.yzu.edu.cn (F.H.); mz120241769@stu.yzu.edu.cn (Z.W.); 2 Institute of Veterinary Immunology & Engineering, National Research Center of Engineering and Technology for Veterinary Biologicals, Jiangsu Academy of Agricultural Sciences, Nanjing 210014, China; jiehongying@jaas.ac.cn (H.J.); hejiahao@stu.njau.edu.cn (J.H.); 20170953@jaas.ac.cn (X.Z.); 20130990@jaas.ac.cn (Y.Z.); 3 Institute of Animal Husbandry & Veterinary Science, Shanghai Academy of Agricultural Sciences, Shanghai 201106, China; jiaojiajie@saas.sh.cn; 4 China-South Africa Joint Laboratory For Prevention and Control of Major Animal Diseases, Nanjing 210014, China; 5 GuoTai (Taizhou) Center of Technology Innovation for Veterinary Biologicals, Taizhou 225300, China

**Keywords:** freeze-drying, animal vaccine, quality of lyophilization animal vaccines, process optimization

## Abstract

Vacuum freeze-drying, commonly referred to as lyophilization, is one of the primary processes in vaccine industrial manufacturing. It offers significant advantages, including a marked extension of the shelf-life of dried products, effective preservation of biological activity, and user convenience. However, in the production of animal freeze-dried vaccines, the diverse nature of antigens, the complexity of heat-stable protectant formulations, and variations in manufacturing equipment pose substantial challenges to process design for animal freeze-dried vaccines. Commercially available animal freeze-dried vaccines commonly suffer from antigen potency loss, insufficient product stability, low production efficiency and high manufacturing costs. Freeze-drying process optimization can markedly improve the quality of freeze-dried vaccines, with key evaluation indicators including vaccine stability, shelf-life, sample morphology, and residual moisture content. Focusing on multiple types of animal vaccines, this review summarizes research advances in improving the quality of animal freeze-dried vaccines from the perspectives of modifying material properties, enhancing freezing-stage regulation, increasing the specific surface area for drying, and optimizing heat conduction. Finally, future research strategies for animal freeze-dried vaccines are prospected. Although numerous approaches have been proposed to enhance the quality of animal freeze-dried vaccines, comprehensive considerations from multiple dimensions are still required to provide more holistic theoretical support for cost reduction and efficiency improvement in the production of animal freeze-dried vaccines.

## Simple Summary

Lyophilization is a well-established technique for the long-term preservation of live vaccines, offering advantages such as superior protection and ease of transportation. However, the quality of lyophilized animal vaccines remains inconsistent, and their widespread application is limited due to high economic costs, the complexity of formulation design, and incompatibility between lyophilization protocols and vaccine solutions. We elaborate on the fundamental principles and procedural steps of the freeze-drying process. Synthesizing the research findings and experience of our team and the broader field, we enumerate the critical factors that determine the quality of lyophilized animal vaccines. This paper aims to elucidate the preparation principles, current status, application value, and future perspectives of lyophilized animal vaccines, with the aspiration of promoting the high-quality application of freeze-drying technology in the field of animal vaccine manufacturing.

## 1. Introduction

Vacuum freeze-drying, commonly known as lyophilization, is a modern, advanced drying technology. First invented by Leslie of the United Kingdom in 1813, this technique was successfully applied by Shackell in 1909 for the preservation of antitoxins, bacterial strains, rabies viruses, and other biological products, yielding favorable results [1]. Freeze-drying technology can effectively preserve bioactive substances, significantly extend the shelf life of dried products, maintain favorable biological stability, and facilitate convenient storage and transportation [2]. Accordingly, it has become one of the core manufacturing processes for biological products. In the field of veterinary vaccines, lyophilization is predominantly applied in the preparation of live attenuated vaccines. Despite the aforementioned advantages of freeze-drying technology, the quality of freeze-dried vaccines is restricted by economic costs. Semi-finished animal vaccine products cannot undergo multi-step purification processes, while the compatibility design of thermostable freeze-drying protectants is highly complicated. In addition, most conventional freeze-drying procedures fail to fully match the physicochemical properties of vaccine solutions, resulting in the generally inferior quality of commercial animal freeze-dried vaccines [3]. The common quality defects include a severe bioactivity loss during freeze-drying, short shelf life, and excessive residual moisture content. These limitations further hinder the industrialization of freeze-drying preservation technology for novel vaccines such as mRNA vaccines and restrict the widespread application of existing animal freeze-dried vaccines. Optimization of freeze-drying processes to improve freeze-drying efficiency is conducive to enhancing the overall quality of animal freeze-dried vaccines, with core evaluation indexes covering product stability, shelf life, cake morphology, and residual moisture content. For instance, cake collapse or excessive residual moisture may trigger antigen degradation and the loss of biological activity [4,5]. Addressing the above technical bottlenecks requires targeted improvement of the freeze-drying efficiency for animal vaccines, and the optimization of these key parameters can further promote the application of vacuum freeze-drying technology in veterinary vaccine production. This review systematically summarizes the research progress of process optimization in the field of animal freeze-dried vaccines and prospects future research strategies for quality improvement, aiming to facilitate the high-quality application of freeze-drying technology in animal vaccine manufacturing.

## 2. Principle of Preparation and Storage Conditions of Animal Freeze-Dried Vaccines

Water exists in three physical states: solid, liquid, and gas. According to thermodynamic principles, the boiling point and freezing point of water vary with ambient pressure. Under reduced pressure, the boiling point and freezing point of water converge, allowing solid ice to sublime directly into gaseous water vapor upon heating. The fundamental mechanism of freeze-drying relies on the phase transition of water under low-temperature and low-vacuum conditions. Specifically, the materials are first frozen at a low temperature, followed by heating under vacuum, which enables ice to sublime directly and eliminates moisture from the samples, ultimately yielding low-temperature dehydrated products [6]. Most commercial live animal vaccines are manufactured via vacuum freeze-drying. Typically, vaccine antigens are mixed with excipients and subsequently processed through vacuum freeze-drying to obtain final dried formulations [7]. Current freeze-dried live animal vaccines are widely applied in poultry, swine, ruminants, waterfowl, and companion animals [7]. Nevertheless, most freeze-dried live vaccines exhibit poor thermal stability and require storage conditions below −15 °C, while vaccines stable at 2–8 °C have not been widely popularized [8]. The primary limitation lies in the technical barriers hindering the industrial transformation of thermostable freeze-drying protocols, mainly due to the insufficient rational design and optimization of freeze-drying procedures [9]. In addition, strict cold-chain transportation substantially increases logistical costs. In regions with inadequate infrastructure, complete cold-chain maintenance cannot be guaranteed, which further restricts the popularization and application of animal freeze-dried vaccines worldwide [10,11]. Accordingly, improving the overall quality and thermal stability of freeze-dried vaccines is critical for achieving cost reduction and efficiency enhancement in the production of live animal freeze-dried vaccines.

## 3. Common Issues in the Development and Industrialization of Lyophilized Animal Vaccines

Currently, although lyophilization have been adopted for veterinary vaccines manufacturing, several technical bottlenecks still remain, which compromise the development efficiency and the quality of freeze-dried veterinary vaccines. First, freeze-drying formulation design is highly complex: different antigens require distinct protectant components, and the screening of suitable protectant formulations is time-consuming [12]. Second, freeze-drying cycles must be well-matched to sample properties. It is necessary to guarantee vaccine quality while shortening drying duration as much as possible to cut energy consumption and production costs. Third, significant equipment gaps exist between laboratory-scale freeze-dryers and large-scale industrial units. Numerous challenges emerge during process scale-up, making it difficult to maintain product homogeneity in industrial production [13,14]. For instance, encouraging progress has been made in the research of veterinary mRNA vaccines [15,16], suggesting that safer and more efficient vaccines can be developed in the future. However, thermostable storage of mRNA vaccines remains a critical bottleneck to overcome [17]. Although the lyophilization of mRNA vaccines is under active investigation [18,19], it is still far from large-scale industrial application.

We have summarized the above challenges in Table 1. The key to addressing these issues lies in the rational optimization of lyophilization processes, thereby facilitating the further development and application of lyophilized animal vaccines.

## 4. Enhancing the Quality of Freeze-Dried Vaccines Through Process Improvement

**Figure 1:**
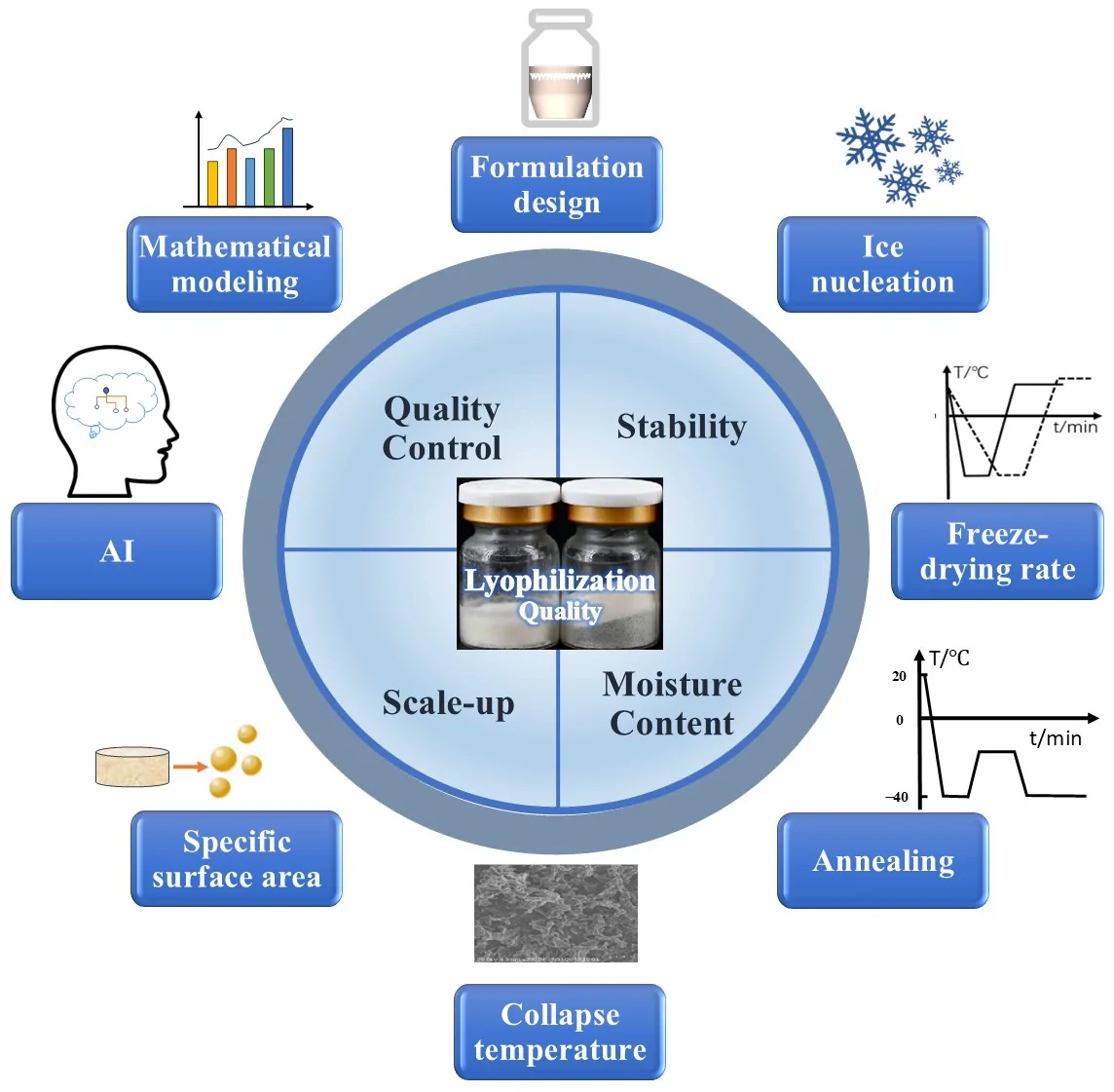
Various factors affecting the quality of freeze-dried animal vaccines.

The key to improving the quality of lyophilized vaccines lies in minimizing antigen damage to preserve vaccine titer. Every stage of the lyophilization process impacts the stability and residual moisture content of the final product. Critical factors include the lyophilization formulation design, the physicochemical properties of the feedstock (such as the eutectic point and collapse temperature), the ice nucleation process, the lyophilization protocol, and the specific surface area of the material being dried (Figure 1). This section briefly outlines strategies for improving lyophilization efficiency based on the aforementioned factors.

### 4.1. Enhancing Stability by Modifying Lyophilized Material Properties

#### 4.1.1. Selecting Appropriate Excipients

Excipients are essential components of lyophilized materials. An acceptable appearance is one of the key quality control criteria for lyophilized vaccines. The selection of appropriate excipients helps maintain an intact cake structure without collapse [31]. A stable cake appearance can mitigate structural damage to active substances during lyophilization and reduce the resultant potency loss of vaccines. Meanwhile, it also facilitates the long-term stable storage of lyophilized vaccines [8]. Key considerations for excipient selection include their chemical composition, concentration, pH value [32], and potential species-specific sensitivities in target animals. Different excipients can provide distinct auxiliary effects for freeze-dried materials. The common excipient components and their functional performances are summarized in Table 2.

Single excipients such as skimmed milk powder and gelatin were commonly used as stabilizers, enabling long-term storage of live lyophilized vaccines at −15 °C. However, recent literature has demonstrated that thermostable protectants composed of multi-component formulations (e.g., sugars, polyols, proteins, gelatin, and amino acids) can preserve the activity of live classical swine fever vaccines for 1–2 years at 2–8 °C [43]. Although some commonly used excipients, such as gelatin, carboxymethyl cellulose, and exogenous proteins, exhibit favorable stabilizing effects, they may induce immune side effects in animals, which can impair vaccination efficacy and even result in vaccine failure [44,45]. Lyu et al. [46] developed a gelatin- and protein-free live classical swine fever vaccine using materials such as dextran and polyvinylpyrrolidone (PVP). This novel formulation extended the shelf-life of the vaccine at 2–8 °C to 30 months (compared to 24 months for commercial products), while simultaneously improving vaccine potency, thermostability, and safety without causing adverse reactions in swine. In addition, the compatibility of excipient components and their concentrations should also be taken into consideration. Kang et al. [47] demonstrated that the combination of 2% sorbitol and 0.5% gelatin applied as excipients for a freeze-dried duck viral hepatitis vaccine exerted superior effects on enhancing the stability and thermal tolerance of vaccine products compared with conventional disaccharide-based excipients. This formulation effectively extended the shelf-life of the vaccine from two weeks to 11 months. Nevertheless, excessive addition of sorbitol (8%) was found to reduce vaccine potency. Furthermore, Wang et al. [48] reported that a high proportion of trehalose in the freeze-dried matrix could inhibit the complete crystallization of mannitol.

#### 4.1.2. Increase the Collapse Temperature of the Vaccine Solution

The collapse temperature refers to the critical temperature at which a lyophilized product loses rigidity and undergoes phenomena similar to collapse or melting during the drying process [49]. When the product temperature reaches or exceeds the collapse temperature, the increased fluidity of the lyophilized matrix disrupts the molecular microstructure, causing direct damage to active substances, which macroscopically manifests as the collapse of the lyophilized product [50]. Elevating the product’s collapse temperature can reduce the lyophilization duration, decrease energy consumption, enhance the drying rate, and improve the stability of lyophilized products [49]. Mannitol is a common excipient used to increase the collapse temperature of solutions [51]. Based on the optimized heat-resistant protectant formula A for classical swine fever virus, our research team further modified the formulation by adding materials such as mannitol and glycine, raising the collapse temperature from −31 °C to −24 °C. This modification resulted in a viral titer loss of only 0.32 ± 0.03 lg after storage at 37 °C for 10 days and successfully shortened the lyophilization time from 42 h to 24 h [43]. Flood et al. [52] demonstrated that formulations supplemented with glycine and sucrose exhibited good morphological stability at 37 °C during the development of thermostable influenza subunit vaccines. These findings indicate that increasing the collapse temperature of the lyophilized material is beneficial for enhancing the stability of the final product and shortening the drying cycle.

### 4.2. Mitigating Potency Loss by Enhancing Freezing Stage Control

The freeze-drying process primarily consists of three stages: pre-freezing, primary drying, and secondary drying [53]. During pre-freezing, the sample temperature is reduced below the freezing point to form a solid state. Ice crystal formation during freezing induces mechanical damage, which is a major cause of potency loss in lyophilized vaccines [34,54]. The size and morphology of ice crystals directly determine the extent of such mechanical damage. Due to ice crystal formation being unavoidable in freeze-drying, controlling the ice nucleation and growth process is critical for minimizing mechanical damage. Any temperature fluctuation after ice formation can trigger recrystallization. Therefore, the pre-freezing temperature, freezing rate, and primary drying temperature are key parameters that dictate ice crystal size and morphology, thereby influencing the sublimation efficiency during primary drying [55]. In the field of animal vaccine freeze-drying, enhancing control during the freezing stage is a proven strategy to improve product stability, shorten processing times, and optimize cake morphology. This can be achieved through three main approaches: controlling ice nucleation [56,57], regulating the freezing rate [58], and implementing temperature annealing [59].

#### 4.2.1. Regulating Ice Crystal Nucleation

The process of ice crystal formation initiates with ice nucleation. Uncontrolled ice nucleus growth exerts mechanical damage on antigenic structures [60], and studies have confirmed that the ice nucleation temperature influences the frozen structure and the collapse temperature of lyophilized products [61]. Controlling ice nucleation enables the modulation of ice crystal morphology, thereby mitigating associated damage and reducing sublimation resistance during primary drying [62,63]. Singh et al. [64], building upon the ice nucleation control method reported by Geidobler [65], demonstrated that controlling ice nucleation during lyophilization enhances the stability of protein lyophilized products and shortens processing time. Meng et al. [56] formulated several ice crystal regulators containing polyhydroxyl structures, which effectively reduced ice crystal size to 25% of the original. After five freeze–thaw cycles at −20 °C, the viral titer loss was maintained below 0.3 lg. Subsequent optimization of the lyophilization protocol yielded a maximum lyoprotective rate of over 82.75%. Compared to the standard lyophilization protocol for commercial products, the optimized process improved lyophilization efficiency by 31.4%. Zuo et al. [57] found that trehalose significantly inhibits ice crystal growth: incorporating 5% trehalose reduced the average ice crystal size of the viral solution to 32.7% of that in the pure viral solution. The average ice crystal size exhibited an inverse relationship with trehalose concentration (5–15%). At a 15% trehalose concentration, the freeze-drying loss of classical swine fever freeze-dried vaccines was minimized to less than 0.1 lg.

#### 4.2.2. Controlling the Freezing Rate

The freezing rate determines ice crystal size, which further affects the drying duration of products as well as the process design space for freeze-dried vaccines [66]. Rapid freezing (cooling at 10~15 °C per min) generates smaller ice crystals within the lyophilized matrix, whereas slow freezing (cooling at less than 1 °C per min) results in the formation of larger ice crystals [67]. Numerous studies have demonstrated that the freezing rate impacts the potency of lyophilized vaccines. Wang et al. [58] compared the effects of slow versus rapid freezing during the optimization of the freeze-drying cycle for Orf virus vaccines. Their results showed that the slow freezing method led to a viral titer loss of 0.5 lg, while the rapid freezing method minimized the loss to only 0.3 lg. Furthermore, the rapidly frozen vaccines exhibited lower residual moisture content and superior reconstitution properties. Similarly, Zuo et al. [43] optimized the energy-efficient lyophilization cycle for thermostable classical swine fever live vaccines. They found that rapid freezing resulted in a titer loss of only 0.17 lg, compared to 0.65 lg with slow freezing, demonstrating that rapid freezing is more suitable for the preparation of freeze-dried vaccines against certain animal-susceptible viruses.

#### 4.2.3. Annealing

Annealing refers to a post-freezing treatment process in which the fully frozen material is heated to a temperature just below the eutectic point, held for a specific duration, and then cooled back to the freezing temperature [68]. This process promotes ice crystal rearrangement, reduces variations in ice crystal size, and modulates ice crystal morphology and size to achieve uniformity, thereby improving cake appearance and enhancing drying efficiency [68]. The underlying mechanism is that smaller ice crystals melt faster than larger ones during annealing; consequently, larger ice crystals absorb smaller ones [54]. This phenomenon leads to an increase in internal porosity, which reduces the mass transfer resistance during ice sublimation [69]. Studies have shown that a simple freezing step sometimes fails to achieve complete crystallization of certain excipients such as mannitol or glycine, which impairs the storage stability of freeze-dried samples. In contrast, annealing can promote the crystallization of the frozen matrix [70]. Zhao et al. [59] investigated three different pre-freezing methods—rapid freezing, slow freezing, and annealing—for the lyophilization of duck hepatitis live vaccines containing sucrose, gelatin, glycerol, and phosphate buffers. Their results indicated that vaccines lyophilized via slow freezing exhibited shrinkage, suggesting insufficient stability. In contrast, vaccines prepared by rapid freezing and annealing formed acceptable cake-shaped products. Specifically, the viral titer loss of vaccines processed with annealing was less than 1.0 lg, demonstrating satisfactory vaccine efficacy.

### 4.3. Reducing Residual Moisture by Increasing Drying Specific Surface Area

Residual moisture content is a critical parameter for ensuring the potency of lyophilized vaccines. Increasing the specific surface area of the lyophilized material is one of the effective methods to reduce residual moisture. Studies have confirmed a strong correlation between residual moisture content and the collapse temperature mentioned earlier [71,72]. Notably, different freeze-dried products possess their own optimal moisture-content ranges, and excessive drying can also undermine the stability of lyophilized products [73]. To reduce residual moisture content, novel freeze-drying technologies have been developed. For instance, Meyer et al. [74] investigated the spin-freezing design. In the spin-freezing approach, vials loaded with freeze-drying materials are rapidly rotated along their longitudinal axis during freezing. The freeze-drying material spreads and forms a thin layer on the inner sidewall of the vial, yielding a larger surface area compared with conventional freeze-dried cake. Experiments demonstrated that for specific products, spin-freezing technology could reduce moisture by 4- to 8-fold, and the drying time by 10- to 40-fold. Meulewaeter et al. [75] validated the feasibility of this approach using a lipid nanoparticle (LNP) model. Drying times of less than 10 h were achieved, with the mRNA-LNP lyophilizates exhibiting no significant changes in particle size or polydispersity index post-drying. The mRNA encapsulation efficiency remained above 80%, and the cellular transfection efficiency of the lyophilized mRNA-LNP was approximately 97% of the liquid control, with successful expression observed in mice. Currently, mRNA vaccines still require cryogenic storage, and their development is hindered by cold chain logistics [17]. This technology can provide support for the research and development of lyophilized mRNA-LNP veterinary vaccines.

### 4.4. Improve the Homogeneity of the Product During the Scale-Up Lyophilization Process by Controlling Heat Transfer Discrepancy

Multiple technical challenges frequently emerge during the scale-up of freeze-drying processes from the laboratory-scale to industrial production, mainly including equipment limitations, inconsistent product temperature distribution, and variable drying durations [76]. These discrepancies primarily stem from differences in heat transfer characteristics, incomplete product drying, equipment geometric features, as well as variations in the scale and condensing capacity of different freeze-dryers. Such issues can lead to unqualified vaccine products, significant batch-to-batch variability, and increased energy consumption, ultimately resulting in raw material waste and elevated production costs [77].

The most prevalent influencing factor is the edge effect during freeze-drying preparation, which refers to the temperature discrepancy across different regions of a single freeze-dryer shelf. Samples placed at the shelf edges receive more heat and achieve more sufficient drying compared with those located in the central area, causing prominent differences in heat exposure and drying degree among samples from the same batch [78]. Furthermore, vials with different materials and specifications, as well as variable filling volumes adopted in industrial vaccine production, can also induce uneven heat distribution in samples [79,80]. Mitigating the edge effect facilitates efficient thermal energy utilization and improves the batch stability of freeze-dried vaccines. Traditionally, freeze-drying process scale-up verification relies heavily on the trial-and-error method, which is extremely time-consuming and material-costly. Currently, numerous researchers have developed innovative strategies to achieve efficient verification of freeze-drying scale-up. Pikal et al. [81] established a theoretical algorithm for calculating the heat transfer coefficients of edge and central vials. This method enables accurate quantification of the edge vial effect, predicts variations in edge effect under different dryers and processing conditions, and optimizes the scale-up procedure of freeze-drying processes, with verified computational accuracy. Zhou et al. [29] determined vial heat transfer coefficients and cake resistance from experimental data based on freeze-drying models. The vial heat transfer coefficients obtained via this approach were consistent with those calculated by the classical gravimetric method. Compared with conventional strategies that acquire key parameters through steady-state freeze-drying model simulation, this method is more convenient, time-saving, and cost-effective. Bernadette et al. [82] constructed a three-dimensional mathematical model correlating freeze-dryer design parameters with heat transfer variations, serving as a powerful tool for freeze-drying process scale-up. As an efficient analytical platform, this model can predict heat transfer discrepancies between edge and central vials within a single device during process design and scale-up, and also enable comparative analyses of heat transfer characteristics among different freeze-drying equipment.

### 4.5. Enhancing Quality Control of Lyophilized Vaccines During Storage by Mathematical Modeling and Artificial Intelligence

Multiple factors can affect the quality of finished freeze-dried vaccine products during storage. Issues such as improper storage temperatures or equipment malfunctions can lead to vaccine failure. Recent studies have proposed mathematical modeling approaches, such as retrospective analysis and Bayesian inference, to predict vaccine stability [83,84]. Yakobi et al. [85] explored the potential of integrating artificial intelligence (AI) with stability prediction models. This hybrid approach allows for the incorporation of multi-dimensional data to achieve higher accuracy and enables the rapid construction of complex vaccine stability models, laying the groundwork for future development. Sonali Das et al. [86] pointed out that few existing studies have comprehensively explored the combined application of modules including digital formulation design, AI-driven development, and geospatial modeling in vaccine storage-and-distribution strategies. Particularly in conflict zones, disaster-stricken areas, and regions severely affected by climate change, the deployment of smart logistics and real-time monitoring for vaccine storage and transportation is critically important. Researchers argue that future advances in freeze-drying will increasingly rely on an integrated framework of mechanistic knowledge, multimodal characterization, AI-assisted decision support, and product-relevant validation, covering both process development and formulation design [87].

### 4.6. Achieve Efficient Production and Preparation of Animal Lyophilized Vaccines During Manufacturing Process by Adjusting Process Design

**Figure 2:**
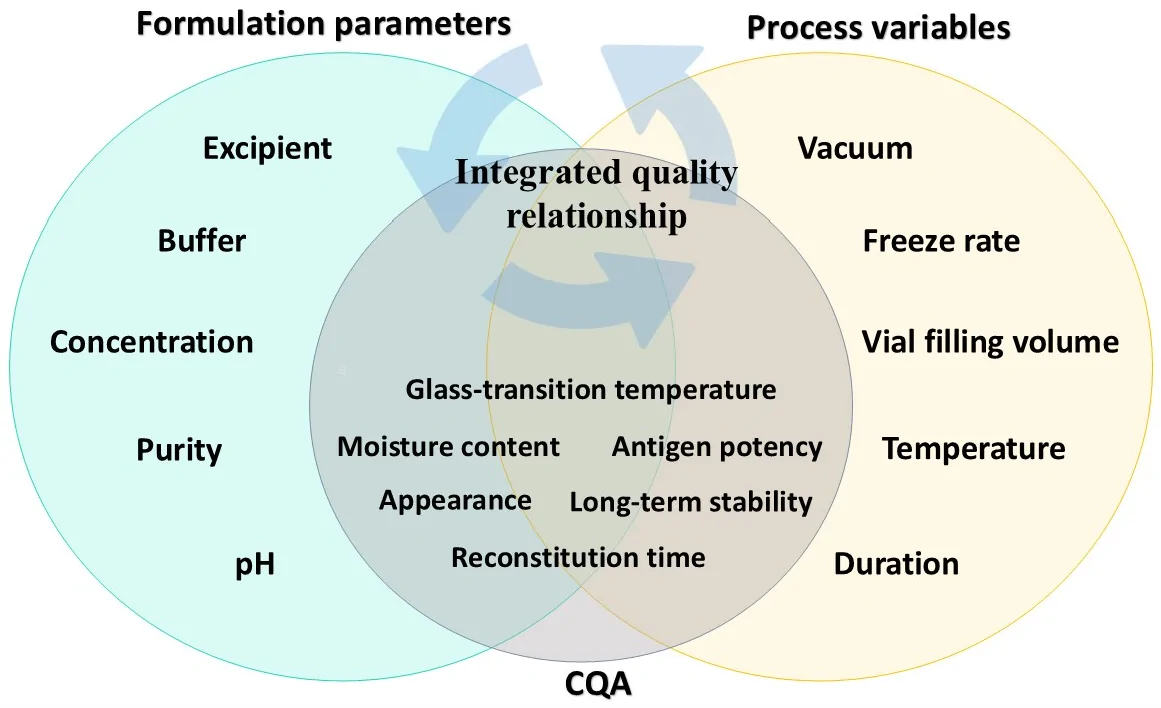
Inter-relationships among formulation parameters, process variables and critical quality attributes for freeze-dried vaccines.

To date, the Quality by Design (QbD) concept has been widely recognized in the pharmaceutical and vaccine industry. The implementation of QbD in freeze-dried vaccine manufacturing aims to reduce product variability and defects. By establishing robust formulations and manufacturing procedures in combination with standardized regulatory management, the efficiency of product development and industrial production can be significantly improved [88,89]. The core components of QbD include prior knowledge accumulation, risk assessment, mechanistic modeling, design of experiments (DoE), data analysis, and process analytical technology (PAT) [88]. The application of QbD in vaccine freeze-drying encounters unique challenges that differ from those in conventional pharmaceutical formulations. These discrepancies mainly originate from the complex biochemical structural characteristics of diverse vaccine antigens, as well as the more intricate manufacturing workflows involved in freeze-dried vaccine production [90]. In Figure 2, we illustrate the mutual relationships among selected formulation parameters, process variables and critical quality attributes (CQAs) involved in the design of freeze-dried vaccines. Nevertheless, numerous emerging strategies have been proposed to extract in-depth information from existing experimental datasets to optimize product design. Several studies have attempted to optimize critical quality attributes of freeze-dried vaccines based on characteristic parameters. Relevant research [91] analyzed the critical quality attributes of freeze-dried vaccines by monitoring the glass-transition temperature, enabling accurate prediction of glass-transition behavior under different excipient concentrations. This approach allows the precise screening of optimal excipient compositions and dosage proportions. Zubak et al. [92] evaluated a newly developed near-infrared (NIR) spectroscopic method for the quantification of residual moisture in freeze-dried meningococcal vaccines. The results demonstrated that the established model could accurately determine residual moisture content ranging from 1.0% to 6.7%. Moreover, comprehensive proportional error verification confirmed that the NIR method exhibited higher accuracy than conventional reference methods, indicating its great potential as a preferred technique for evaluating intra-batch and inter-batch variability as well as the stability of freeze-dried vaccines.

In summary, although multiple strategies are available to improve the overall quality of animal freeze-dried vaccines, comprehensive consideration of various restrictive factors is still required. First, extensive resources are still consumed in formulation and process validation, and sufficient experimental data are essential to verify the equivalence or superiority of modified vaccine formulations compared with conventional products [93]. In addition, the incomplete definition of immune protection correlates for numerous veterinary pathogens, together with inconsistent regulatory criteria across different regions and authorities, further hinders industrial upgrading. Other practical limitations include insufficient laboratory infrastructure and limited detection manpower [94]. The introduction of the “One Health” concept into the field of animal freeze-dried vaccines can promote the coordinated advancement of veterinary vaccine quality, animal health management, and public health safety [95].

## 5. Conclusions and Future Perspectives

With the development, application and industrialization of novel veterinary vaccines represented by mRNA-LNP vaccines, higher standards have been raised for lyophilization technology. The quality of final veterinary vaccine products is directly determined by core technical optimizations, including rational formulation design to alleviate drying-induced structural damage and enhance vaccine stability, as well as precise regulation of ice nucleation, recrystallization and ice crystal morphology to reduce freezing damage to vaccine antigens.

In the case of mRNA-LNP vaccines, lyophilization is theoretically compatible with the technical requirement of preventing mRNA hydrolysis during storage. However, ice crystals formed in the freezing stage can easily rupture LNP structures during growth, leading to the leakage of encapsulated mRNA molecules and ultimately impairing vaccine efficacy [54]. In addition, protective excipients may produce osmotic concentration gradients during freezing, which further induces payload leakage from mRNA-LNP nanoparticles [96]. Early studies in 2007 have verified that lyophilization can effectively improve the stability of naked mRNA molecules [97]. Although no lyophilized mRNA vaccine has been commercially available so far, numerous studies have successfully applied lyophilization strategies to mRNA-LNP vaccine preparation [18,98], fully demonstrating the technical feasibility and promising prospect of lyophilized mRNA vaccine formulations.

This review systematically summarizes and discusses the latest progress in lyophilization process optimization of veterinary freeze-dried vaccines, aiming to provide a theoretical basis and technical support for quality improvement in veterinary vaccine lyophilization. Despite the remarkable advances in the quality optimization of veterinary freeze-dried vaccines in recent years, there remains considerable room for further research and innovation in the future: (1) explore optimal pre-freezing rates and temperatures for different types of antigens to establish customized freezing parameters with minimal antigen damage; (2) develop more efficient control strategies for the freezing stage to significantly improve overall drying efficiency and production economy; (3) accelerate the exploration and applicability verification of innovative lyophilization technologies, such as continuous lyophilization and thin-film lyophilization, in the field of veterinary vaccine manufacturing; (4) develop specialized excipient systems tailored to novel nano-formulations including mRNA-LNPs, clarify the interaction mechanisms among lyoprotectants, lipid carriers and nucleic acid molecules, and reduce structural damage from the perspective of molecular thermodynamics; (5) adopt artificial intelligence tools to assist the optimization of lyophilization process analytical technology (PAT), and realize full-process intelligent quality control during the production, storage and transportation of freeze-dried vaccines; and (6) strengthen the industrial supervision of veterinary freeze-dried vaccines and formulate unified and standardized evaluation criteria for novel veterinary lyophilized vaccine products.

In the context of the global energy shortage, improving the thermostability of vaccine products has become an essential design principle for the research and development of modern veterinary vaccines. Enhanced thermal stability is also an urgent demand for expanding vaccine application scenarios and coping with sudden animal disease epidemics. With the joint efforts of researchers in biology, pharmaceutics, food engineering and interdisciplinary fields, vacuum freeze-drying technology will surely achieve further cost reductions, efficiency improvements and high-quality industrial applications in veterinary vaccine manufacturing.

## Figures and Tables

**Table 1:** Common issues and underlying causes in the development and industrialization of freeze-dried animal vaccines.

Issues	Main Causes	References
Complex Formulation Design	(1) Protective agent components require extensive screening (2) Incompatibility between protective agent components and antigens (3) High cost of some protective agent components	[20,21,22,23]
Incompatible Lyophilization Programs	(1) Failure to identify the most suitable lyophilization program (2) Lack of effective monitoring during the lyophilization process (3) Inability to perform real-time adjustments during the lyophilization process	[24,25,26]
Insufficient Uniformity During Lyophilization Process Scale-Up	Equipment differences between laboratory and industrial scales	[27,28]
Excessively High Costs During Lyophilization Process Scale-Up	Substantial costs associated with trial-and-error calibration	[29,30]

**Table 2:** Common excipients and functions applied in freeze-dried vaccines.

Examples	Group	Function	References
Mannitol	Sugar alcohol	Enhances the structural stability of the freeze-dried cake and prevent collapse	[33]
Bovine serum albumin	Protein	Increases the glass-transition temperature of dried samples	[34]
Sucrose	Disaccharide	Occupation of antigen water-binding sites through vitrification and reduction in molecular mobility in dried state	[35]
Trehalose	Disaccharide	Promotion of glass-transition in freeze-dried matrix	[36,37]
Glycine	Amino Acid	Assisted enhancement of freeze-dried matrix structural stability to reduce particle aggregation upon storage	[38,39,40]
Sorbitol	Sugar alcohol	Preserves the native structure of antigens and improves their structural stability	[41]
Gelatin	Natural macromolecule	Increases collapse temperature of the material of interest to get higher drying temperatures	[42]

## Data Availability

No new data were created or analyzed in this study. Data sharing is not applicable to this article.
